# Human infection caused by *Streptococcus suis* serotype 2 in China: report of two cases and epidemic distribution based on sequence type

**DOI:** 10.1186/s12879-020-4943-x

**Published:** 2020-03-14

**Authors:** Fei Jiang, Jingjing Guo, Chen Cheng, Bing Gu

**Affiliations:** 1 Department of Laboratory Medicine, the Affiliated Hospital of Xuzhou Medical University, No.99 West Huaihai Road, Xuzhou, 221002 Jiangsu Province China; 2 Department of Laboratory Medicine, Xuzhou Central Hospital, No.199 South Jiefang Road, Xuzhou, 221009 Jiangsu Province China; 3 Medical Technology School of Xuzhou Medical University, No.209 Tongshan Road, Xuzhou, 221004 Jiangsu Province China

**Keywords:** *Streptococcus suis*, Serotype 2, ST7, Whole-genome sequencing, Case report

## Abstract

**Background:**

*Streptococcus suis* is a zoonotic pathogen that causes serious systemic infections in pigs and occupation-related infections in humans who contact with pigs or pork products. In China, it has caused two outbreaks of human infection and surveillance for *S.suis* has been ongoing since last time.

**Case presentation:**

Two cases of meningitis and sepsis caused by *S. suis* were reported in this study. Both patients work in relation to the pork trade, a risk factor for *S. suis* infection. The outcome was favorable after a prolonged ceftriaxone therapy but one patient was left with mild hearing loss. Two isolates were identified as sequencing type (ST) 7, *S. suis* serotype 2 (SS2), which is one the most prevalent and cause two outbreaks in China. Whole-genome sequencing (WGS) revealed that a high degree identity was noted in the genome organizations and sequences between two sporadic ST7 SS2 isolates in this study and representative epidemic virulent isolates. Major differences among them are two sporadic ST7 SS2 isolates lacked a virulence factor called agglutinin receptor and an 89 K pathogenicity island (PAI), which plays important role in the pathogenesis of streptococcal toxic shock syndrome (STSS). A summary about STs of human infection with *S. suis* in China was completed. The result showed ST1 and ST7 were still the major STs and several novel STs were successfully discovered in different provinces.

**Conclusions:**

Our results enhanced the understanding of the ability to cause life-threatening infections in humans and the distribution and evolution of the *S. suis* in China.

## Background

*Streptococcus suis* is a zoonotic pathogen that causes serious systemic infections in pigs and occupation-related infections in humans who contact with pigs or pork products. The most common clinical manifestations are meningitis and sepsis. Globally, serotype 2, ST1 isolates have been described as mostly responsible for *S. suis* human infection, particularly in South America, Europe, Japan, Southeast Asia and China [1]. Serotype 2, ST7 isolates were another predominant sequence type and responsible for the 1998 and 2005 Chinese epidemics, which caused a total of 240 human infections, 52 of whom dead [2]. Surveillance for *S.suis* has been ongoing since last outbreak in our country [3–5]. Here we report two cases of successfully cured human infection with ST7 SS2. Genomic characteristics were investigated by WGS and the epidemic distribution of *Streptococcus suis* isolated from human was summarized based on sequence type in China to better monitoring of this pathogen.

## Cases presentation

In September 2016, a 67-year-old man with an 8-year history of emphysema (case 1) was admitted to the Affiliated Hospital of Xuzhou Medical University because of 2-day history of headache with nausea and fever and 1-day consciousness disturbance. Physical examination revealed neck stiffness and inability to speak, a temperature of 38.7 °C, pulse rate of 110 beats/min, respiratory rate of 20 breaths/min, and blood pressure of 133/80 mmHg. A cerebral computed tomography (CT) scan showed no obvious abnormality. Laboratory result for leucocyte cell count was 12.23 × 10^9^/L (86.4% neutrophils). Lumbar puncture showed turbid cerebrospinal fluid (CSF) with increased white cell numbers (1807 × 10^6^/L, 82.9% neutrophils), high protein levels (2.8 g/L) and low glucose levels (1.77 mmol/L). Blood and CSF were collected for culture before empirical meropenem (2 g IV q8h) were prescribed. After 2 days, blood and CSF cultures showed α-hemolytic *streptococci* which was identified as *S. suis* by MALDI-TOF MS (Bruker Daltonik GmbH, Germany) with a log score 2.369 (above 2.0 is considered reliable for species identification). The patient was diagnosed with purulent meningitis and sepsis. This isolate was susceptible to ampicillin (MIC≤0.25 μg/mL), penicillin (MIC≤0.06 μg/mL), ceftriaxone (MIC≤0.5 μg/mL), vancomycin (MIC≤1 μg/mL), levofloxacin (MIC = 2 μg/mL), linezolid (MIC≤2 μg/mL) and meropenem (MIC≤0.25 μg/mL), but resistant to erythrocin (MIC≥16 μg/mL), clindamycin (MIC≥8 μg/mL), tetracycline (MIC = 32 μg/mL). The patient further reported working as a pork seller. It is a pity that further information regarding whether there was any open wound was not be provided. The antibiotic treatment was replaced by ceftriaxone 2 g IV q12h. The patient was successfully treated with no particular adverse events after 24 days.

A previously healthy 48-year-old man (case 2) with a 1-day history of fever and obnubilation was hospitalized to Xuzhou Central Hospital in July 2017. At admission, physical examination showed impaired consciousness, nuchal rigidity and temperature was 38.4 °C, blood pressure 159/98 mmHg, heart rate 105 beats/min, and respiratory rate 46 breaths/min. CT scan showed bilateral cerebral hemispheres with superficial sulci and cerebral swelling was considered. His leucocyte cell count, procalcitonin and C-reactive protein were 14.89 × 10^9^/L (90.5% neutrophils), 36.42 ng/mL and 199 mg/L respectively. CSF had a leukocyte count of 80 × 10^6^/L, a protein level of 1.3 g/L and a glucose level of 2.16 mmol/L. Blood and CSF culture were performed before empirical application of ceftriaxone (2 g IV q12h). After 3 days, blood and CSF cultures showed α-hemolytic gram-positive *streptococci*. The isolate was identified as *S.suis* by MALDI-TOF MS with a log score 2.230 and showed susceptibility to ampicillin (MIC≤0.25 μg/mL), penicillin (MIC≤0.06 μg/mL), ceftriaxone (MIC≤0.5 μg/mL), vancomycin (MIC≤1 μg/mL), levofloxacin (MIC = 2 μg/mL), linezolid (MIC≤2 μg/mL), meropenem (MIC≤0.25 μg/mL), erythrocin (MIC≤0.25 μg/mL), clindamycin (MIC≤0.25 μg/mL) and only resistance to tetracycline (MIC = 32 μg/mL). After *S. suis* was identified, the patients reported engaging in the cooked food selling as a profession. And before he became ill, he contacted a sick dead pig with a wound on his hand. This patient was diagnosed with purulent meningitis and sepsis and he improved on administration with ceftriaxone 2 g IV q12h for 15 days and methylprednisolone 80 mg IV qd for 4 days, followed by 40 mg IV qd for 7 days and 20 mg IV qd for 4 days. However, pure tone audiology evaluation showed mild bilateral hearing loss (− 40 dB). The patient’s hearing ability was not normalized in 2 months of follow-up after discharge.

**Fig. 1:**
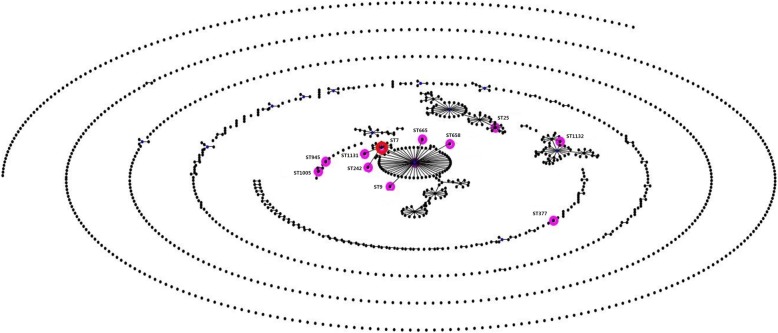
An eBURST analysis of the *S. suis* MLST database (accessed on July 18, 2019). The sequence types found in China was marked with pink circles. ST7 *S. suis* in this study belonged to CC7 (red circle)

**Fig. 2:**
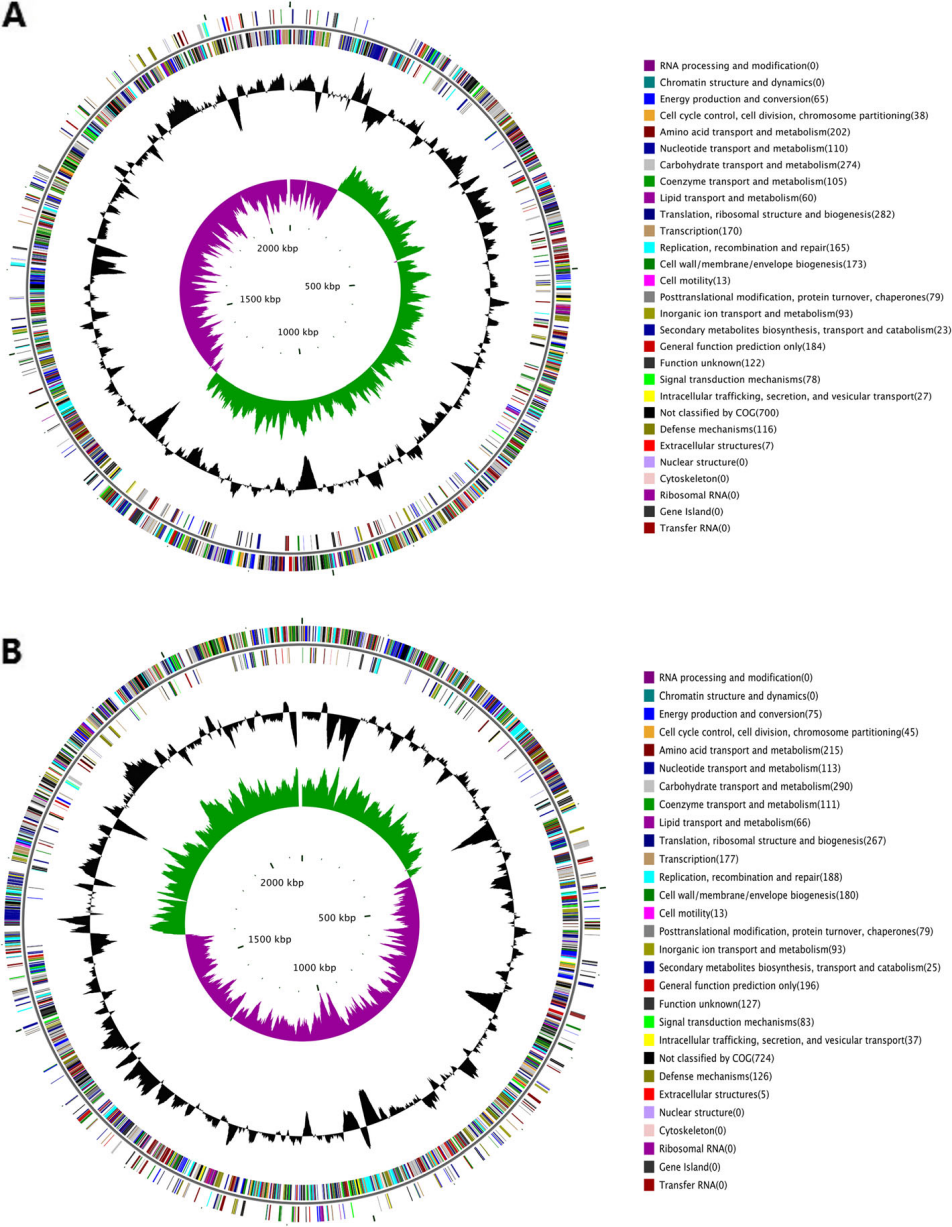
Circular diagrams and in silico comparison of two *S. suis* genomes(*S.suis1* (**a**) and *S.suis2* (**b**)) . From outside to inside, in circle 1, the coding genes of COG outside (inside) represent the genes located on the positive (negative) strand, different colors represent different biological functions. In circle 2 (black circle), the GC contents of the genomes were shown. In circle 3, GC skew [(G-C)/(G + C)] is displayed (Green indicates values> 0, and purple indicates values< 0)

Further analysis was performed to investigate their characteristics of these two isolates. Multilocus sequence typing (MLST) determined that two isolates assigned to ST7, which was the most predominant sequence type in China [1]. On eBURST v.3.0 analysis (Fig. 1), ST7 belongs to the clone complex 7 (CC7) and is a single-locus variant (SLV) of ST1. Two isolates were serotyped by multiplex PCR (a technique that cannot differentiate serotype 2 from 1/2 and serotypes 1 and 14) and confirmed as serotype 2 by WGS data (Fig. 2a, b) and the *S. suis* serotyping pipeline (https://github.com/streplab/SsuisSerotyping_pipeline). The pipeline uses the *cpsK* gene (*Streptococcus suis* NSUI002, GenBank accession number CP011419) missense mutation at position 161 to differentiate serotypes 2 and 1/2.

Comparing to genome of representative epidemic isolates (05ZYH33 and 98HAH33) [3], a high degree identity was noted in the genome organizations and sequences. Two sporadic ST7 isolates carried all virulence factors except agglutinin receptor. In addition, the major difference in genome of two sporadic ST7 isolates in this study was the absence of 89 K PAI specific to epidemic isolates, which play an important role in the pathogenesis of STSS.

**Fig. 3:**
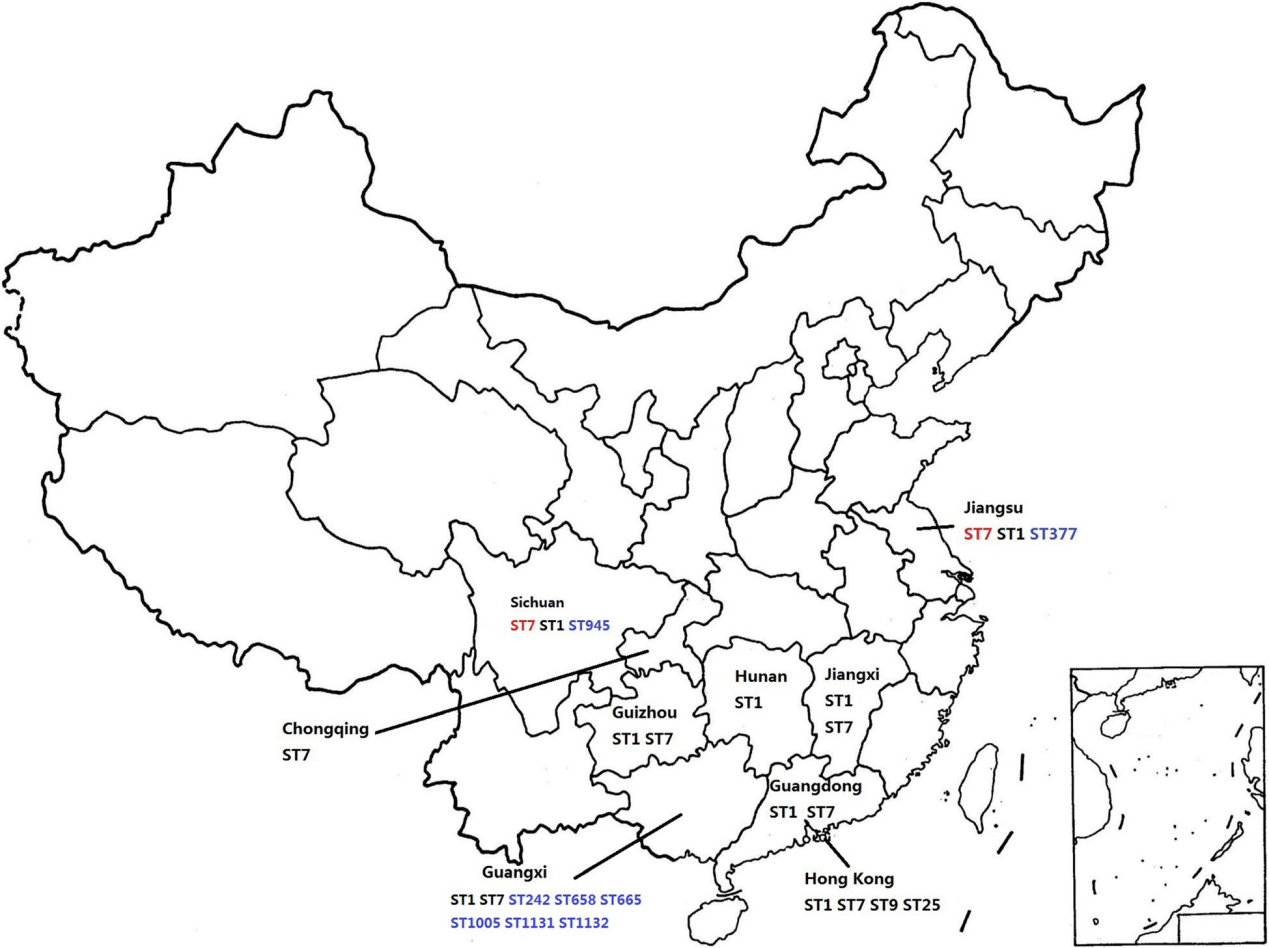
Geographic distribution of human *S. suis* infections based on ST in China. Two large-scale outbreaks caused by ST7 are indicated in red and the newly discovered STs are labeled in blue. All other sporadic cases are presented in black

According to the MLST database (https://pubmlst.org/ssuis) and previous geographic reviews [1, 3–6], we summarized and mapped the geographic distribution of *S.suis* causing human infection in China. The collected epidemiological data showed that *S. suis* cases occur mainly in South China. Except for two large outbreaks occurred in 1998 and 2005, other human infection cases were sporadic infections. ST1 and ST7 were still the main sequence types relevant to *S. suis* human infection in China. Meanwhile, several novel sequence types (ST242, ST377, ST658, ST665, ST945, ST1005, ST1131 and ST1132) were gradually discovered in different provinces in recent years (Fig. 3). Additional concerns should be raised because of the high pathogenicity of these microbes. It is interesting that these newly discovered STs were found exclusively in humans according to MLST database. As a zoonotic pathogen, *S. suis* is primarily and usually found in pigs and there is no direct evidence to date showing human-to-human infection [7]. Therefore, future work should focus on a broader sampling from humans and pigs and comparative analyses between them.

## Discussion and conclusions

Human infection caused by *S. suis* is a rare but life-threatening condition. Epidemic and sporadic human infection cases caused by *S. suis* serotype 1, 2, 4, 5, 9, 14, 16, 21, 24 and 31 have also been reported in many countries and 17.8% of patients dead [1, 8–10].Timely and accurate diagnosis of pathogenic bacteria is the key to cure. Because of the variable of biochemical characteristics, *S. suis* is often misidentified as other species of *Streptococcus* genus [7]. MALDI-TOF MS is a rapid and accurate technique used to detect isolates of *S.suis*. In addition to serum agglutination, specific multiple PCR was developed for serotype analysis, but cannot distinguish serotype 2 from serotype 1/2 [11]. In a recent report, serotype and genotype (multilocus sequence type) of isolates has been demonstrated to serve as predictors of pathotype [12]. Thus, serological evidence or analysis of the *cps* locus based on WGS and ST were essential to investigate the pathogenic pathotype. STs of SS2 were diverse worldwide, such as ST1 and ST147 in Europe, ST1, ST7, ST25 and ST28 in Asia, ST25 and ST28 in North America and ST1 in South America [1]. Obviously ST1 was the most predominant. However, ST7, to which two isolates in this study belong, has caused the 1998 and 2005 outbreaks of human infection relative to sporadic infections caused by ST1 in China [8, 13]. At the same time, newly sequence types were gradually discovered in recent years and additional concern should be raised to prevent potential large-scale spread.

As well-recognized to be a highly virulent, epidemic ST7 SS2 isolates carried lots of virulence factors [14]. An 89 K PAI, correlated with STSS and high pathogenicity to some extent, was confirmed to be prevalent and unique among the extremely virulent SS2 isolates that are responsible for both large scale outbreaks of severe human SS2 infections in China [15]. Through the annotation of virulence factors of pathogenic bacteria, we did not find centralized and continuous virulence genes. At the same time, we compared the assembled results with *S. suit*-related virulence islands and no virulence island region existed in these two isolates. This explains the absence of STSS in our patients. Whether agglutinin receptor plays an important role in the pathogenicity of *S. suis* will be further investigated.

Wound exposure to pigs and pork is considered to be one of the most important ways to contract *S. suis*. Human infections duo to consumption of raw pork products suggest that the gastrointestinal tract is also a major route of entry in cases of *S. suis* infections, especially in patients with damage to the intestinal barrier [16]. In our cases, both case-patients were at risk for infection because their profession was engaged in the pork trafficking, and one patient contacted a sick dead pig with an open wound on his hand, suggesting the source of the infection. Because breakpoints for *S. suis* are not defined in the Clinical and Laboratory Standards Institute guidelines, breakpoints for *Streptococci viridians* were used instead in this study [17]. Results of antibiotic sensitivity test showed that two isolates were sensitive to most antibacterial agents. It is reported that 99.1% *S. suis* was resistant to tetracycline and 67.9% to erythromycin and clindamycin because of the present of *tet* and *erm* resistance genes [18]. We did not perform the detection because erythromycin and tetracycline was not the preferred drug for treating *S.suis* infection. According to the previous literature [19], a prolonged treatment course and close monitoring of patients upon completion of therapy for signs and symptoms of recrudescent disease were suggested and if symptoms recur, an additional treatment course based on clinical response will be considered.

Our results enhanced the understanding of the distribution and evolution of the *S. suis* and their ability to cause life-threatening infections in humans. ST1 and ST7 are still predominant in China and newly STs should also be strict monitored to prevent potential spread.

## Data Availability

All original data used and analyzed during the current study are available from the corresponding author on reasonable request. The datasets generated and analyzed during the current study are available in the CNSA repository. (CNSA project ID: CNP0000908. Accession numbers: CNA0007340 and CNA0007341. https://db.cngb.org/cnsa/project/CNP0000908/reviewlink/).

## Abbreviations

CSF: Cerebrospinal fluid
CT: Computed tomography
MALDI-TOF MS: Matrix-assisted laser desorption ionization-time of flight mass spectrometry
MIC: Minimum inhibitory concentration
PAI: Pathogenicity island
*S. suis*: *Streptococcus suis*
ST: Sequence type
WGS: Whole-genome sequencing
